# Local Electric‐Field‐Driven Fast Li Diffusion Kinetics at the Piezoelectric LiTaO_3_ Modified Li‐Rich Cathode–Electrolyte Interphase

**DOI:** 10.1002/advs.201902538

**Published:** 2019-12-17

**Authors:** Mengting Si, Dandan Wang, Rui Zhao, Du Pan, Chen Zhang, Caiyan Yu, Xia Lu, Huiling Zhao, Ying Bai

**Affiliations:** ^1^ School of Physics & Electronics Henan University Kaifeng 475004 P. R. China; ^2^ National Demonstration Center for Experimental Physics and Electronics Education School of Physics & Electronics Henan University Kaifeng 475004 P. R. China; ^3^ School of Materials Sun Yat‐sen University Guangzhou 510275 P. R. China

**Keywords:** electrochemical performance, interfacial engineering, Li‐rich cathodes, lithium ion batteries (LIBs), piezoelectric LiTaO_3_ coating layer

## Abstract

As one of the most promising cathodes for next‐generation lithium ion batteries (LIBs), Li‐rich materials have been extensively investigated for their high energy densities. However, the practical application of Li‐rich cathodes is extremely retarded by the sluggish electrode–electrolyte interface kinetics and structure instability. In this context, piezoelectric LiTaO_3_ is employed to functionalize the surface of Li_1.2_Ni_0.17_Mn_0.56_Co_0.07_O_2_ (LNMCO), aiming to boost the interfacial Li^+^ transport process in LIBs. The results demonstrate that the 2 wt% LiTaO_3_‐LNMCO electrode exhibits a stable capacity of 209.2 mAh g^−1^ at 0.1 C after 200 cycles and 172.4 mAh g^−1^ at 3 C. Further investigation reveals that such superior electrochemical performances of the LiTaO_3_ modified electrode results from the additional driving force from the piezoelectric LiTaO_3_ layer in promoting Li^+^ diffusion at the interface, as well as the stabilized bulk structure of LNMCO. The supplemented LiTaO_3_ layer on the LNMCO surface herein, sheds new light on the development of better Li‐rich cathodes toward high energy density applications.

## Graphical abstract

The decorated LiTaO_3_ with intrinsic characteristic of stress response at the surface of active LNMCO induces a local electric field, which significantly promotes Li^+^ diffusion at the cathode–electrolyte interphase, particularly in the discharge process. The application of the “initiative” function interphase material herein contributes to the comprehension of interphase engineering in a broad range of applications in the electrochemical and energy conversion community.

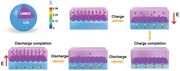

## 1 Introduction

Lithium‐ion batteries (LIBs) have been highly expected in electric vehicles (EVs), hybrid electric vehicles (HEVs) and plug‐in HEVs (PHEVs), besides the traditional applications in portable devices. To build the next generation LIBs with higher performances, high energy density materials are urgently pursued worldwide.^1^, ^2^, ^3^ Lithium‐rich (Li‐rich) materials, with the specific capacity over 260 mAh g^−1^ and energy density up to ≈1000 Wh kg^−1^,^4^ have attracted great interest in the past decades. It is reported that Li‐rich materials are composed of two phases of Li_2_MnO_3_ (*C*_2/m_) and LiMO_2_ ($R\bar{3}m$) (M = Ni, Co, Mn, etc.).^5^, ^6^, ^7^, ^8^, ^9^ Despite the above advantages, several concerns including structural instability and the resulted voltage degradation, as well as the poor diffusion kinetics at the interface have become the bottlenecks of Li‐rich materials.^9^, ^10^, ^11^, ^12^, ^13^

In this regard, multifarious modification approaches, such as doping and surface coating, have been intensively investigated.^14^, ^15^, ^16^ Particularly, Li^+^ diffusion at the cathode–electrolyte interphase (CEI) is widely regarded as the rate determining step in LIBs.^17^, ^18^, ^19^ From this viewpoint, metal fluorides (FeF_3_,^20^ MOF,^21^, ^22^ AlF_3_,^23^, ^24^ etc.), metal oxides (MgO,^25^ Al_2_O_3_,^26^, ^27^ etc.), metal phosphates (AlPO_4_,^28^ LaPO_4_,^29^ Li_3_PO_4_,^30^ FePO_4_/Li_3_PO_4_,^31^ Li‐Mn‐PO_4_,^32^ etc.), and those with similar structure of Li‐rich Li_2_MnO_3_ (Li_2_SiO_3_ 33, 34 and Li_2_SnO_3_,^35^) have been widely applied to modify the surface of bulk Li‐rich materials. Recently, fast lithium‐ion conductors (LiVO_3_,^36^ Li_2_ZrO_3_,^37^ Li‐La‐Ti‐O,^38^, ^39^ LiPON,^40^ etc.) have also been proposed to decorate the surface of Li‐rich cathodes to enhance the apparent diffusion coefficients. All the aforesaid surface modification materials, unexceptionally, have been proved to be effective in both stabilizing the structure and facilitating the Li^+^ kinetics. Nevertheless, in general, the decoration layers themselves seem rather “passive” in promoting Li^+^ diffusion. Assuming they are Li^+^ conductive (e.g., solid electrolyte materials), fast Li^+^ diffusion channels will be provided besides the general separation effect (in suppressing side reactions and inevitable TM dissolution). As for Li^+^ insulators (e.g., metal fluorides), only the benefit of physical barriers could be exploited. Therefore, a more “initiative” function interface is imperative to be built to more effectively promote the Li^+^ transport at the electrode–electrolyte interphase.

It is noteworthy that piezoelectric material, as an important category in the energy‐conversion community, works on the basic conversion law of mechanical energy into electrical one, in which electric field will be induced under pressure.^41^, ^42^ Very recently, piezoelectric family has been added in fabricating composite electrode to boost Li^+^ diffusion kinetics, establishing its peculiar function as an accelerator for Li^+^ migration.^43^ Ramadoss et al. built a piezoelectric PVDF‐ZnO film to drive self‐charging supercapacitor cell, directly converting mechanical energy into electrochemical one.^44^ Wang utilized the piezoelectric PVDF film as a separator to assemble the new‐style self‐charging LIBs, wherein the internal piezoelectric field was believed to promote Li^+^ diffusion.^45^ Lee et al. directly mixed piezoelectric BaTiO_3_ with silicon, then encapsulated them together inside carbon nanotubes, obtaining high Li^+^ conductive compound anode.^46^ The aforementioned piezoelectric materials involved composite electrodes enlighten us to exploiting them as advantageous surface decoration layers. With a piezoelectric coating layer, the deintercalation/intercalation of Li^+^ during charge/discharge will induce continuous respiration of crystal lattice for a specific electrode, thereby exerting an extra stress on the coating layer, which could intrinsically regulates local electric‐field at the electrode–electrolyte interphase.

As a typical piezoelectric material with high thermal stability,^47^, ^48^ LiTaO_3_ (LTO) is believed to be highly compatible with Li‐rich structure due to the slight lattice discrepancy. Thus in this work, LiTaO_3_ is selected as a decoration material to homogeneously encapsulate the Li‐rich cathode material, with the purpose of regulating the surface Li^+^ kinetics through its piezoelectric property and therefore further enhancing the electrochemical performances. As a result, it is established that LiTaO_3_ modification layer could not only accelerate Li^+^ migration at the surface, but also effectively stabilizes the bulk structure of Li‐rich cathode.

## 2 Results and Discussion

**Figure 1:**
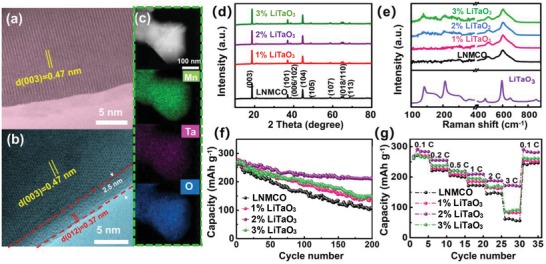
TEM images of a) LNMCO and b) 2% LiTaO_3_ samples; TEM element mapping images of c) 2% LiTaO_3_ sample. d) XRD patterns of the LNMCO, 1%, 2%, and 3% LiTaO_3_ samples; e) Raman spectra of the LNMCO, 1%, 2%, 3% LiTaO_3_ and pristine LiTaO_3_ samples; f) Galvanostatic cycling properties of the LNMCO, 1%, 2%, and 3% LiTaO_3_ samples at 0.1 C. g) Rate capabilities of the LNMCO, 1%, 2%, and 3% LiTaO_3_ samples under different charge–discharge rates.

The four as‐prepared samples display the similar morphologies with an average particle size of ≈250 nm, as investigated by SEM (Figure S1a–d, Supporting Information). And no prominent difference could be observed from the surface morphologies, implying that the modification layers may be actually very thin. Lattice fringes with the *d*‐spacing of 0.47 nm could be distinguished in bare and 2% LiTaO_3_ in high‐resolution transmission electron microscopy (HRTEM) observations (**Figure** **1**a,b), corresponding to the (003) plane of Li‐rich material. However, a ≈2.5 nm surface region with a *d*‐spacing of ≈0.37 nm appears for the 2% LiTaO_3_ sample, which could be indexed to the crystallized LiTaO_3_ layer growing along (012) direction. From the view of defect, the lattice mismatch in Figure S3a,b (Supporting Information) is calculated to be as low as 3.1% (shown in Table S1, Supporting Information), indicating a desirable interfacial compatibility between the decorated LiTaO_3_ and bulk LNMO material. Moreover, the electron dispersive spectroscopy (EDS) mappings from SEM (Figure S4, Supporting Information) and TEM (Figure 1c) demonstrate the uniform distributions of O, Ta, Co, Mn, and Ni elements at low and high spatial resolutions.

X‐ray diffraction (XRD) patterns of the bare and LiTaO_3_‐Li_1.2_Ni_0.17_Mn_0.56_Co_0.07_O_2_ (LNMCO) samples are shown in Figure 1d. All the peaks could be indexed to α‐NaFeO_2_ type hexagonal structure of the space group $R\bar{3}m$ .^49^ Besides, a bimodal feature of the (006)/(012) and (018)/(110) peaks at ≈38^o^ and 65^o^ are prominent, providing indicators of typical layered structure.^50^ In addition, the weak peaks between 20° and 25° could be observed, in accordance with the superlattice of monoclinic unit cell *C_2/m_*, which are attributed to the Li/Mn ordering in Li_2_MnO_3_.^49^ In order to evaluate the antisite of Li/Ni ions and the layered structure of as‐prepared samples, the ratios of *I*_(003)_/*I*_(104)_ and *c/a* are calculated^51^ and shown in Table S2 (Supporting Information). It is obvious that all samples demonstrate the layered structure with a low cation mixing, among which 2% LiTaO_3_ sample displays the least Li/Ni antisite. For comparison, bare LiTaO_3_ is also prepared through the same procedure, and its XRD pattern (Figure S5a, Supporting Information) agrees well with the pure target material (JCPDS#29‐0836). However, no diffraction peaks of LiTaO_3_ are found in the XRD curves for 1%, 2%, and 3% LiTaO_3_ samples, which could be attributed to the low contents, since they could be clearly observed when the proportion increases up to 10 wt% (Figure S5b, Supporting Information). According to Rietveld refinement (Figure S6, Supporting Information), the lattice parameters experience little difference after decorated by LiTaO_3_ (Table S2, Supporting Information), suggesting that the intrinsic structure of LNMCO bulk is not affected by LiTaO_3_ coating layers.

Raman spectra of the pristine and LiTaO_3_‐LNMCO samples are provided in Figure 1e. Prominent vibrations at 447 and 594 cm^−1^ could be assigned to the typical $R\bar{3}m$ group, corresponding to the A_1g_ (stretchings of Ni^2+^—O, Mn^3+^—O, Mn^4+^—O) and *E*_g_ (Co^3+^—O) modes.^52^, ^53^, ^54^ In addition, the weak peak at around 421 cm^−1^ is indexed to the monoclinic structure of *C*_2/m_ from the vibration of Li_2_MnO_3_.^52^, ^55^ Although the Raman bands of LiTaO_3_ cannot be observed in the pristine and 1% LiTaO_3_ samples, those peaks located at ≈150, 200, and 600 cm^−1^ gradually appear when the coating content reaches to 2 wt%. Further fitting results demonstrate the vibration signals of Ta‐O in all the modified samples (Figure S7, Supporting Information), confirming the successful deposition of LiTaO_3_ on the surface of Li‐rich material.

X‐ray photoelectron spectroscopy (XPS) analysis provides more evidence of the successful LiTaO_3_ surface modification on LNMCO particles. Figure S8 (Supporting Information) demonstrates the XPS spectra of all the as‐prepared samples, in which Ta signal could be clearly observed in the LiTaO_3_‐decorated samples, which is absent in pure LNMCO material. Specifically, the binding energies of Ta 4f orbitals are found located at ≈25.80 and 27.95 eV, consistent with the Ta^5+^ ion in LiTaO_3_. It should also be noted that the signal of Ta increases along with the augment of LiTaO_3_ coating content (Figure S8b, Supporting Information). Meanwhile, the binding energies of Mn 2p, Ni 2p, and Co 2p (Figure S8d–f, Supporting Information) orbitals, to show the presence of Mn^3+^, Mn^4+^, Ni^2+^, and Co^3+^ ions, continuously decrease in intensities with the enhanced Ta signal. The above findings provide strong evidence of the successful LiTaO_3_ surface modification on LNMCO particles. Fitting analysis of the high‐resolution Mn 2p spectra (Figure S9, Supporting Information) reveals that the Mn^4+^/Mn^3+^ ratios augment from 1.35 to 2.41 with the increased LiTaO_3_ content, which could potentially suppress the Jahn‐Teller effects in Li‐rich cathodes.^56^, ^57^

The initial galvanostatic cycling profiles, as compared in Figure S10 (Supporting Information), display similar characteristics of typical Li‐rich material. More careful observation indicates that the 2% LiTaO_3_ electrode exhibits both the elevated discharged potential and increased specific capacity. As shown in Figure 1f, capacity retentions of the LNMCO, 1, 2, and 3 wt% LiTaO_3_ samples are 41.9% (109.1 mAh g^−1^), 54.0% (141.3 mAh g^−1^), 80.3% (209.2 mAh g^−1^), and 55.6% (148.0 mAh g^−1^) after 200 cycles at 0.1 C, respectively. Obviously, the 2 wt% LiTaO_3_ electrode exhibits the best cyclability among all the electrodes. This electrode also shows a large initial coulombic efficiency (ICE) of 76.8%, higher than those of the LNMCO (70.1%), 1% LiTaO_3_ (71.2%), and 3% LiTaO_3_ (69.4%), respectively. Moreover, the rate performance of 2% LiTaO_3_‐modified sample again, exhibits the best capability under different rates (Figure 1g), which delivers a larger capacity of 172.4 mAh g^−1^ at a high rate of 3 C with respect to that of pure LNMCO (50 mAh g^−1^). From another aspect, the voltage decay upon cycling is one of the main bottlenecks in the practical application of Li‐rich cathodes, which is well‐acknowledged due to the phase transition from the layered structure to the spinel type.^11^ In this case, the voltage decay of 2% LiTaO_3_ sample is ≈0.109 V after 50 cycles, much superior to that of the LNMCO (0.324 V), as shown in Figure S11 (Supporting Information). The above electrochemical characteristics suggests that the LiTaO_3_ modification layers contribute to the improved electrochemical properties, and the 2% LiTaO_3_ electrode is established to be the most effective.

CV curves of the bare and 2% LiTaO_3_ samples within the initial two cycles are presented in Figure S12a,b (Supporting Information). Two distinct oxidation peaks appear at ≈4.0 and ≈4.5 V in the initial profile, corresponding to oxidations of Ni^2+^/Co^3+^, and O loss in Li_2_MnO_3_, respectively.^58^ In the delithiation process, two reduction peaks could be observed at ≈3.25 and 3.7 V, which could be assigned to the reductions of Mn^4+^ and Ni^4+^/Co^4+^ ions, respectively.^54^ Meanwhile, the full width at half maximum (FWHM) values of the oxidation peaks (denoted as O1, O2, and O3) as well as their redox polarizations are calculated and compared in Table S3 (Supporting Information). Apparently, the 2% LiTaO_3_ modified electrode exhibits smaller FWHM and D‐values compared with the pure LNMCO, implying that LiTaO_3_ decoration helps in enhancing the ionic diffusion kinetics.

**Figure 2:**
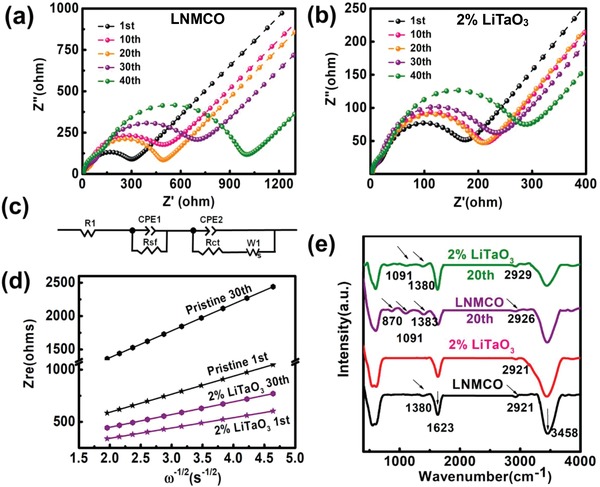
Nyquist plots of a) pristine LNMCO and b) 2% LiTaO_3_ during cycling; c) the equivalent electric circuit for in situ EIS. d) *Z*_re_ versus ω^−1/2^ plots in the low‐frequency region obtained from in situ EIS; e) ex situ FTIR spectra of the pristine LNMCO and 2% LiTaO_3_ samples before and after 20 cycles.

To further investigate the Li^+^ diffusion kinetics of LNMCO cathode after modification, in situ electrochemical impedance spectroscopy (EIS) spectra of the pristine and 2% LiTaO_3_ electrodes are measured at fully delithiated state after charging for 48 h to reach equilibrium. As depicted in **Figure** **2**a,b, the Nyquist plots are consisted of two semicircles and an inclined low‐frequency tail, which are further fitted by the equivalent circuit shown in Figure 2c. R1 represents Ohmic resistance corresponding to the intercept in the high frequency range, which arises from all electrode components including electrolyte, electrode material and separator. And the high‐frequency semicircle reflects the resistance of CEI film (*R*_sf_). The medium frequency semicircle is linked to the charge transfer resistance (*R*_ct_) in the space charge layer. Moreover, the inclined line in the low frequency range is attributed to the Warburg impedance *W*_1_, reflecting the solid‐state diffusion of Li^+^ ions in the bulk electrode material.

According to Figure 2a,b, it is visualized that the semicircle diameters of the pristine cathode increase more prominently than those of the 2% LiTaO_3_, suggesting the underlying contribution to accelerate the interfacial Li^+^ diffusion from the LiTaO_3_ modification layer. Moreover, the fitted *R*_s_, *R*_sf_, and *R*_ct_ (Table S4, Supporting Information) values further evidence the advantages of LiTaO_3_ modification layer in both suppressing the CEI growth and promoting the interfacial charge transfer process.

The apparent diffusion coefficients (*D*_Li_) of LNMCO with/without LiTaO_3_ modification are then estimated from the EIS profiles in the low‐frequency zone.^30^ As listed in **Table** **1**, the analysis of *D*_Li_ directly confirms that the 2 wt% LiTaO_3_ modification layer contributes to the enhanced Li^+^ diffusion kinetics upon cycling. To further clarify this result, the exchange current density is calculated by using the following Equation (1) to evaluate the dynamic behavior of Li^+^

(1) $$i_{0}=\frac{RT}{nFR_{\text{ct}}}$$

wherein *R*_ct_ is the charge transfer resistance, *F* is the Faraday constant, *T* is the absolute temperature, *n* is the electron number involved in the reaction, and *R* is the gas constant. The exchange current densities of the pristine and 2% LiTaO_3_ samples were calculated during different cycles. At 1st cycle, the exchange current densities of the two electrodes are determined to be 4.19 × 10^−5^ and 1.54 × 10^−4^ A cm^−2^, respectively, which degrade to 2.38 × 10^−5^ and 7.51 × 10^−5^ A cm^−2^ after 40 cycles. Obviously, the LiTaO_3_ coating layer effectively enhances the exchange current density, thus leading to excellent Li^+^ diffusion kinetics behavior.

**Table 1:** Apparent diffusion coefficients of Li^+^ for the bare and 2% LiTaO_3_ at 1st and 40th cycles

	LNMCO 1st	2% LiTaO_3_ 1st	LNMCO 40th	2% LiTaO_3_ 40th
Σ	172.82	98.87	402.09	122.07
*D*_Li_ [cm^2^ s^−1^]	1.12 × 10^−10^	3.42 × 10^−10^	2.07 × 10^−11^	2.25 × 10^−10^

Next, the direct current internal resistance (*R*) is used to evaluate the polarization of electrode material,^59^, ^60^ which could be calculated using the following formula

(2) $$R=\frac{V_{t}-V_{0}}{I}$$

wherein *I* is the charge/discharge current, *V_t_* is the voltage at the time of *t* during charge/discharge process, and *V*_0_ is the initial voltage. Besides, the charge voltage is denoted as positive, and the discharge potential is labeled as negative. It is well‐acknowledged that larger *R* value indicates the severer voltage decay and the worse electrode performance. As seen in Figure S13 (Supporting Information), it is clear that the *R* value for 2% LiTaO_3_ material is stabilized at a lower level in comparison with that of the pristine electrode upon cycling. This confirms the small polarization for 2% LiTaO_3_ material, which from another viewpoint explains the boosted Li^+^ kinetics and electrochemical properties after LiTaO_3_ modification.

The above‐mentioned degradation in Li^+^ diffusion upon cycling is strongly related with the interfacial side reactions, e.g., the growth of CEI film.^64^, ^65^, ^66^, ^67^, ^68^, ^69^ In this regard, the ex situ Fourier transformed infrared (FTIR) spectra of the pristine and 2% LiTaO_3_ electrodes are collected before and after 20 cycles. It is clear that the new appeared peaks could be mainly assigned to CEI components for both electrodes (**Table** **2**). Since the vibration signals were obtained from different electrode materials, their intensities could not be compared directly. The collected curves were first normalized according to the invariable M—O bonds (M = Mn, Ni, Co) before they were compared in Figure 2e. Obviously, the peak intensities of CEI film in LNMCO are stronger than those of the 2% LiTaO_3_, demonstrating that the LiTaO_3_ layer could effectively protect the active electrode from side‐reactions with the liquid electrolyte. As a result, the electrochemical performances are improved remarkably and the thermo‐resistance is also enhanced as shown in Figure S14 (Supporting Information).

**Table 2:** New peaks in FTIR spectra and their corresponding assignments

Peak position [cm^−1^]	Assignment
870	CO_3_^2−^ bend, Li_2_CO_3_ 61
1380, 1383	COO^−^ bend, HCOOLi^61^
1623	C=O asym stretching, RCOOLi^62^
2921, 2926, 2929	C—H, ROCO_2_Li^63^
3458	H—O—H, trace amounts of adsorption water^63^

**Figure 3:**
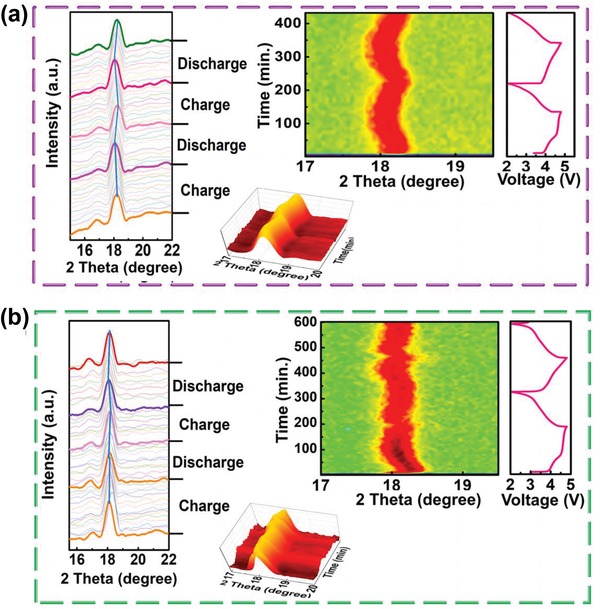
In situ XRD spectra for a) LNMCO and b) 2% LiTaO_3_‐LNMCO and the magnified details of (003) peaks during the same process.

To check the influence of LiTaO_3_ modification layer on the structural evolution, the in situ XRD is performed on both the pristine and 2% LiTaO_3_ electrodes during cycling. **Figure** **3** demonstrates the collected in situ XRD patterns during the first two cycles. Apparently, the shift of (003) peak directly reflects the elongation and shrinkage of lattice constant ***c***, which also implies the underlying phase transition upon Li^+^ insertion and extraction in Li‐rich material. In Figure 3a,b, the swing of (003) peak becomes obviously slighter for 2% LiTaO_3_‐modified electrode compared with that in pristine LNMCO upon cycling, indicating the stabilization in crystal structure of LNMCO after LiTaO_3_ modification, which will consequently improve the capacity retention as shown in Figure 1f.

**Figure 4:**
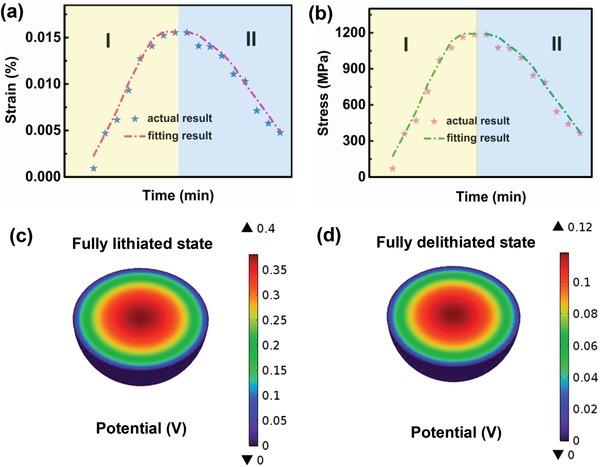
a) Strain and b) stress images of the 2% LiTaO_3_ sample during charge and discharge. The simulation results of the stress piezoelectric potential for the pure LiTaO_3_ material at fully c) lithiated and d) delithiated states.

As aforementioned in the in situ XRD characterization, the interior strain will be introduced accompanying with the respiration of lattice constant, the release of which will be inevitably imposed on the LiTaO_3_ decoration layer to generate local electric‐field due to the piezoelectric property. The improved electrochemical performance confirms that this attached electric field helps in accelerating the interfacial Li^+^ diffusion kinetics. In order to quantitatively elucidate the local potential contributed by LiTaO_3_ layer, the nonlinear dynamic variation of the interior strains from in situ XRD (**Figure** **4**a) are first analyzed by Jade 6.0, followed by evaluating the corresponding stresses through Equation (3)

(3) σ=E⋅ε

wherein *E* is the elastic coefficient (Young's modulus) of the LNMCO material (as provided in Table S5, Supporting Information), and σ and ε values stand for the corresponding stress and strain, respectively. Thus obtained stress (Figure 4b) clearly increases gradually during the charge process and reaches its summit at high cutoff voltage. Inversely, the stress decreases continuously in the discharge process and drops down to its minimum when this process is completed. The fluctuated stress will be transferred to the piezoelectric coating layer of LiTaO_3_, inducing the corresponding local potential. Apparently, the larger the stress is, the higher the piezoelectric potential will be generated. Therefore, it is necessary to simulate the piezoelectric potential at the end of charge and discharge to survey the whole electrochemical process by COMSOL Multiphysics 5.2a. As a result, the voltage direction remains opposite to that of stress upon cycling, with internal piezoelectric potentials varying from 0 to 0.36 V and from 0.36 to 0.12 V during delithiation and lithiation processes (Figure 4c,d), respectively.

**Figure 5:**
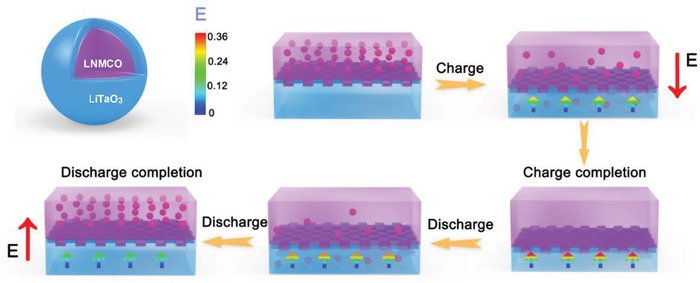
The dynamic and piezoelectric scheme for the LiTaO_3_‐modification layer of the LiTaO_3_‐LNMCO material during one charge–discharge cycle.

**Figure** **5** diagrams the role of piezoelectric coating layer under the electrochemical environment. During the charge process, the oxidization of the O—O bonds is enhanced with Li^+^ extraction, followed by inevitable lattice expansion, leaving the coating layer subjected to stress. With the continuous extraction of Li^+^, the increasingly enhanced stress stimulates higher and higher local potential with opposite direction of Li^+^ motion. In this stage, the detrimental overcharge could be alleviated to some extent. As for the discharge process, the stress begins to be weakened as the lattice recovered, which is still expanded compared with the original state, thus the directions of stress as well as local potential (in alignment with the external electric field) remain unchanged. It should be emphasized that the local electric field generated by piezoelectric effect is consistent with the discharge orientation, which will intrinsically accelerate the Li^+^ diffusion, especially at the interface of LNMCO electrode.

## 3 Conclusion

In this work, typical piezoelectric material of LiTaO_3_ is employed to decorate the surface of LNMCO to boost its electrochemical performances. Intensive in situ and ex situ characterizations combined with theoretical simulation reveal that the enhanced electrochemical properties should be ascribed to the LiTaO_3_ coating layer, which not only serves as an “initiative” accelerator in promoting Li^+^ diffusion from its intrinsic piezoelectric property, particularly at the cathode–electrolyte interphase in discharge process, but also enhances the structure stability upon cycling as a robust protection layer. Employing the unique piezoelectric property of LiTaO_3_, this work provides an effective and facile strategy to artificially regulate and optimize the Li^+^ kinetics at the cathode electrolyte interphase, which contributes to the comprehension of interphase engineering in a broad range of applications in the electrochemical and energy conversion community.

## 4 Experimental Section

*Synthesis of Li_1.2_Ni_0.17_Mn_0.56_Co_0.07_O_2_ Sample*: Layered LNMCO was synthesized through a simple sol‐gel method. In a typical experiment, stoichiometric precursors of lithium acetate (Kermel), manganese acetate (Aladdin), nickel acetate (Aladdin) and cobalt acetate (Aladdin) were separately dissolved in the same solvent of ethyl alcohol at room temperature first. With vigorous stirring, the acquired solutions were dropped into the citric acid (Kermel) solution (with metal cations: C_6_H_8_O_7_ = 2:3). Then, the mixture was evaporated at 80 °C until a light‐purpled sol was formed, which was afterward aged at 120 °C for 12 h to acquire the corresponding gel. Next, the gel was collected out and ground adequately in a mortar to obtain fine powder, which was then calcined at 450 °C for 10 h to remove organic contents. Finally, thus collected powder was ground carefully and reannealed at 900 °C for 12 h to obtain the target material.

*Preparation of LiTaO_3_‐Modified LNMCO Samples (LTO‐LNMCO)*: First, Ta_2_O_5_ (Ourchem) and CH_3_COOLi (Kermel) powders with the same content of 0.05 mol were separately dispersed/dissolved into ethyl alcohol in two flasks with the same full scale volume of 50 mL, controlling the concentration to be 1 mol L^−1^. Then, various contents of Ta_2_O_5_ and CH_3_COOLi solutions with the volume ratio of 1:2 were extracted from the two flasks and mixed by vigorous stirring. Next, the as‐prepared pristine LNMCO powder was homogeneously dispersed into the above mixture. Afterward, the obtained solution was evaporated at 100 °C followed by annealing at 500 °C for 48 h to obtain the target products. The actual contents of LiTaO_3_ were determined to be 0.72, 1.86, and 2.63 wt% for the 1, 2, and 3 wt% LiTaO_3_‐coated LNMCO by inductively coupled plasma spectroscopy (ICP) analysis. For simplicity, the nominal 1%, 2%, and 3% LiTaO_3_ were used hereafter.

*Physical Characterizations*: The crystal structures were detected by using XRD (DX2700, 40 KV) equipment, assembled with Cu‐Kα radiation of λ ≈ 0.15418 nm scanning from 10^o^ to 110^o^ with a scanning step of 0.026^o^ s^−1^. Raman spectra (Renishaw RM‐1000) were detected to analyze the composition of as‐prepared samples with 633 nm He‐Ne laser. XPS was used to analyze the chemical environment on the thermo electron corporation spectrometer with Al Kα radiation source (1486.6 eV). The morphologies and element distributions of the as‐prepared samples were imaged on a field emission scanning electron microscope (FESEM, JEOL 7001F) with the accelerating voltage of 10.0 kV, equipped with EDS. The TEM and HRTEM images with electron EDS were collected on an FEIG2F20 instrument to survey the samples. FTIR measurements were performed on the Nicolet AVATAR360 Fourier‐transformed infrared spectrometer. Differential scanning calorimetry (DSC, TA Q600) was adopted to explore the thermostabilities of electrodes, in which the hermetical cathode was heated to 480 °C at a rate of 5 °C min^−1^ in Ar. The elasticity modulus was evaluated on a commercial atomic force microscope (AFM) dimension icon (Bruker, Santa, CA, USA), with a probe type of RTESPA‐525 (spring constant *K* = 200 N m^−1^). The proportion of each element in the samples was determined by ICP (Perkin Elmer Optima, 2100DV). A furnace of KSL‐1200 (HF‐Kejing, China) was selected to perform heat treatment in this work. The heating rate was constantly controlled to be 2 °C min^−1^, and the natural cooling process was always adopted.

*Electrochemical Measurements*: The powders of as‐prepared samples (as active cathode materials), acetylene black (as conductive agent) and PVDF (as binder) were mixed with a weight ratio of 8:1:1. Then the mixture was thoroughly ground, followed by dissolving in *N*‐methyl‐2‐pyrrolidone (NMP) to form a homogeneous slurry. Next, the mixed slurry was uniformly spread onto a piece of aluminum (Al) foil, and then dried at 100 °C for 12 h. Subsequently, the dried foil was cut into square sheets with a size of 8 mm × 8 mm as electrodes. The coin cells (CR2032) were assembled in an argon‐filled glove box, with Li foil as counter electrodes and Calgary 2400 porous polypropylene film as separators. The electrolyte was composed of 1 m LiPF_6_ in ethylene carbonate and dimethyl carbonate with the volume ratio of 1:1. The cells were measured on the NEWARE test system (Shenzhen, CT‐2001A, China), which were operated at the current densities from 0.1 to 3 C (1 C = 376 mA g^−1^) within the voltage range from 2.0 to 4.8 V (vs Li/Li^+^). Cyclic voltammetry (CV) was tested on the Chenhua CHI660D electrochemical workstation with a scan rate of 0.2 mV s^−1^. The EIS was detected on the same electrochemical workstation over a frequency range of 100 kHz to 5 mHz with a voltage amplitude of 5 mV.

## Conflict of Interest

The authors declare no conflict of interest.

## Supporting information

Supporting Information
