# Supplementary material for: Local Electric‐Field‐Driven Fast Li Diffusion Kinetics at the Piezoelectric LiTaO3 Modified Li‐Rich Cathode–Electrolyte Interphase
Source: Adv Sci (Weinh). 2019 Dec 17;7(3):1902538. doi: 10.1002/advs.201902538 (PMC7001634; doi:10.1002/advs.201902538)
Supplement: Supplementary file 1 — Supporting Information [file ADVS-7-1902538-s001.pdf]

Copyright WILEY-VCH Verlag GmbH & Co. KGaA, 69469 Weinheim, Germany, 2019.

ADVANCED SCIENCE
Open Access

Supporting Information

for *Adv. Sci.,* DOI: 10.1002/advs.201902538

Local Electric-Field-Driven Fast Li Diffusion Kinetics at the Piezoelectric $LiTaO_3$ Modified Li-Rich Cathode–Electrolyte Interphase

Mengting Si, Dandan Wang, Rui Zhao, Du Pan, Chen Zhang, Caiyan Yu, Xia Lu, Huiling Zhao, and Ying Bai**

Local Electric-field-driven Fast Li Diffusion Kinetics at the Piezoelectric $LiTaO_3$ Modified Li-rich Cathode-electrolyte Interphase

Mengting Si, [a] Dandan Wang, [a] Rui Zhao, [a] Du Pan, [a] Chen Zhang, [a] Caiyan Yu[a, b], Xia Lu[c], Huiling Zhao [a, *], Ying Bai [a, *]

[a] School of Physics & Electronics, Henan University, Kaifeng 475004, P.R China.

[b] National Demonstration Center for Experimental Physics and Electronics Education, School of Physics & Electronics, Henan University, Kaifeng 475004, P.R China.

[c] Sun Yat-sen University, Guangzhou 510275, P.R. China.

*Corresponding authors. Tel.: +86-0371-23881602; E-mail addresses: zhao@henu.edu.cn, ybai@henu.edu.cn

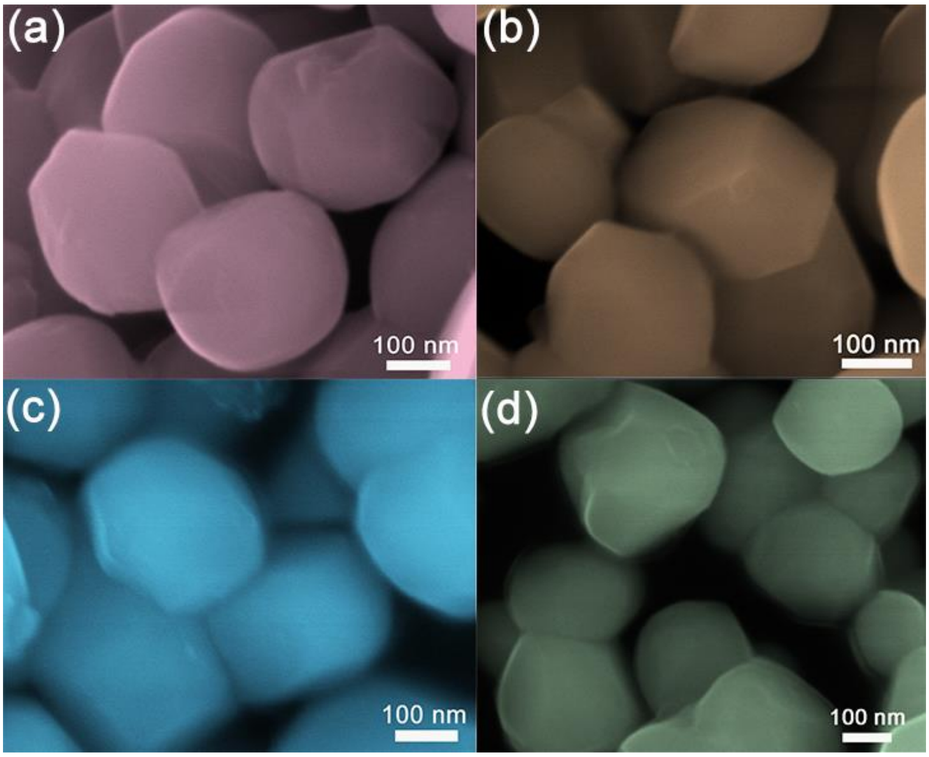

Figure S1 SEM images of **(a)** LNMCO and **(b-d)** 1 %, 2% and 3% $LiTaO_3$ samples.

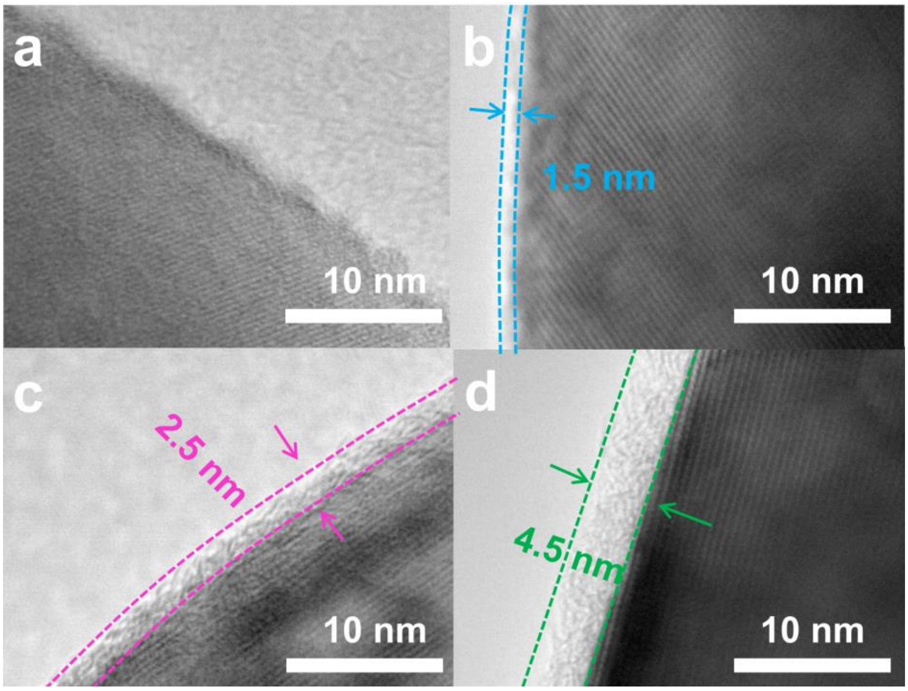

Figure S2 TEM images of **(a)** LNMCO and **(b-d)** 1 %, 2% and 3% $LiTaO_3$ samples.

To distinguish the surface morphologies among LNMCO and the modified samples, TEM (JEM-2100PLUS) images were collected to compare the thickness and homogeneity of all the as-prepared samples (**Figure S2**).

Compared with LNMCO, uniform layers are unexceptionally observed on the surface of $LiTaO_3$-modified samples. Though the decoration layers are generally thin, which are hard to be probed by SEM, they are homogeneously distributed outside the LNMCO particles with increased thickness as the $LiTaO_3$ content enhances from TEM observations.

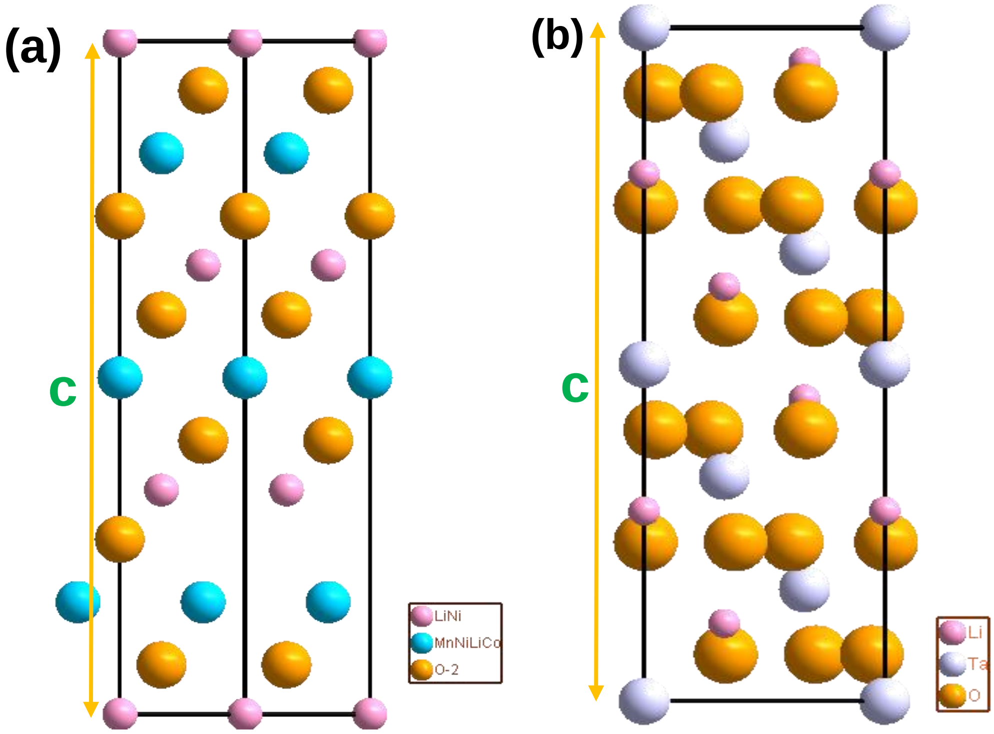

Figure S3 Diagrams of the **(a)** LNMCO and **(b)** $LiTaO_3$ crystal structures.

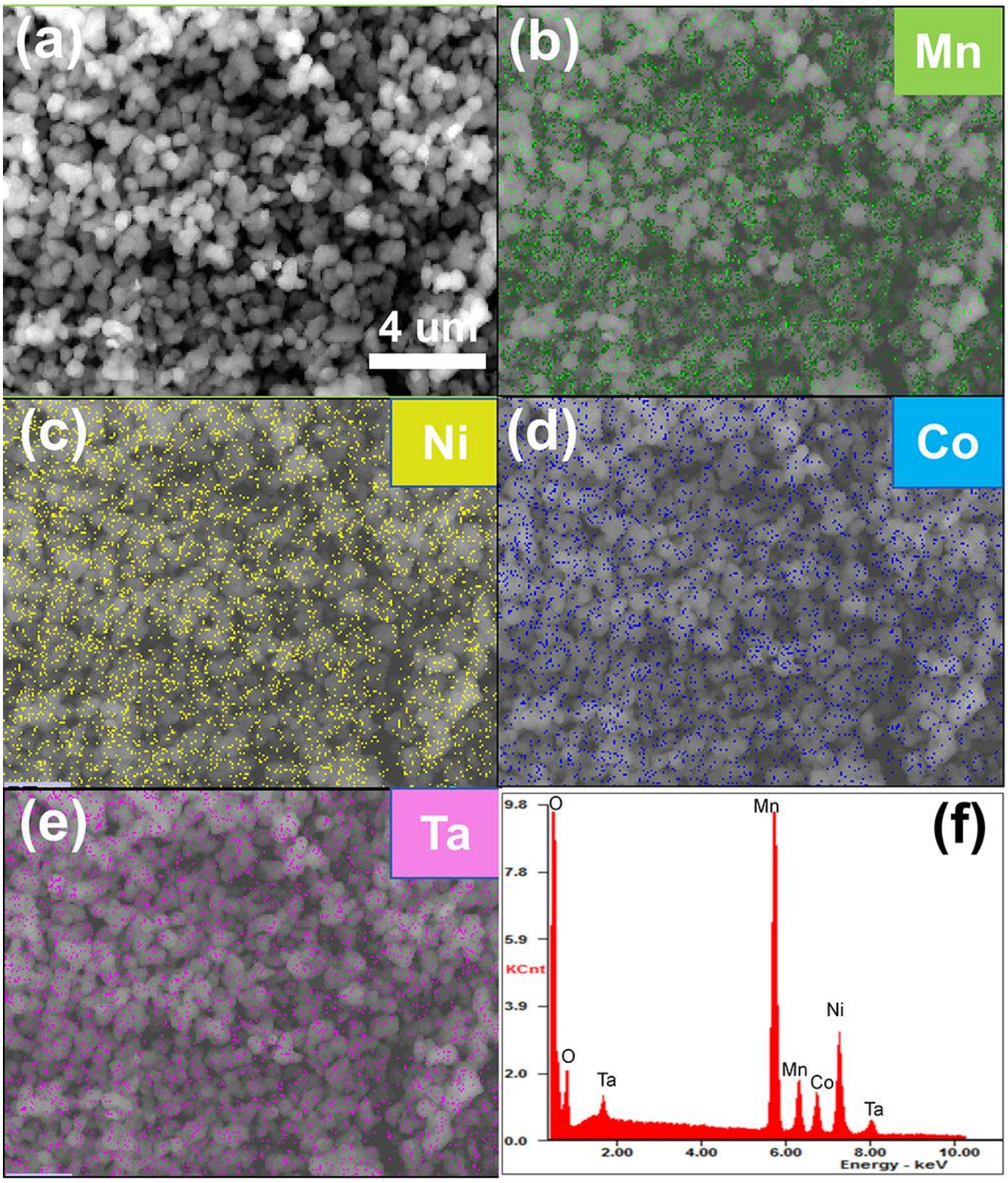

Figure S4 (a) SEM image and the **(b-e)** element mappings as well as **(f)** EDS spectrum for the 2 % $LiTaO_3$ sample.

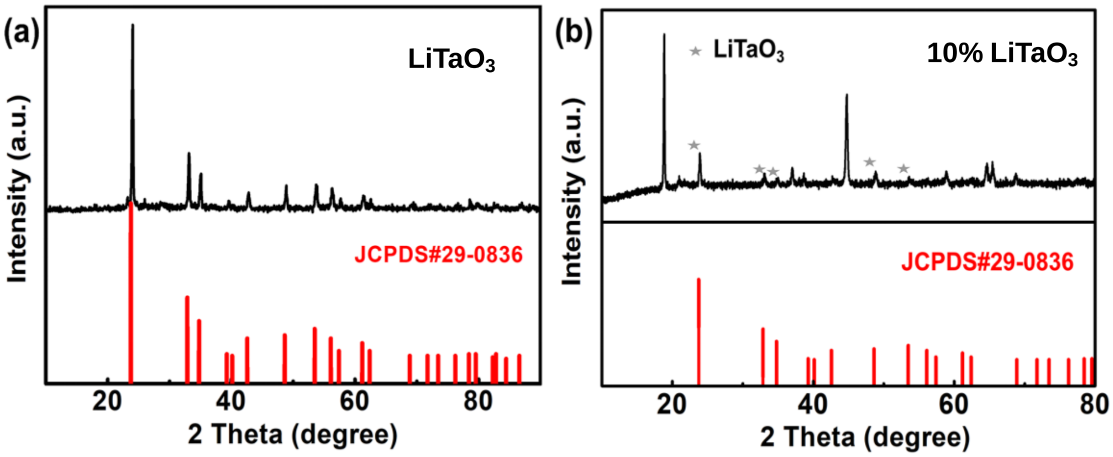

Figure S5 XRD patterns of the **(a)** $LiTaO_3$ and **(b)**10 % $LiTaO_3$.

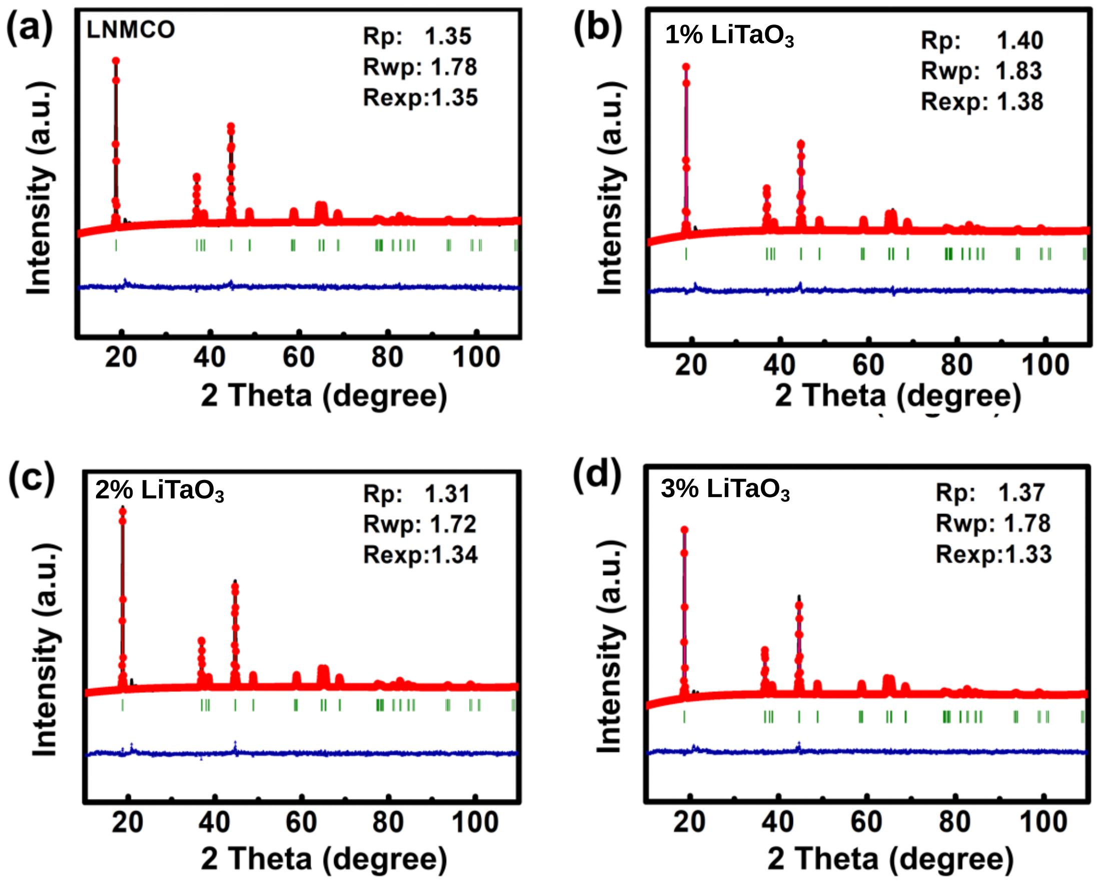

Figure S6 Rietveld refinement XRD spectra of **(a)** LNMCO, **(b)**1%, **(c)** 2% and **(d)** 3% $LiTaO_3$

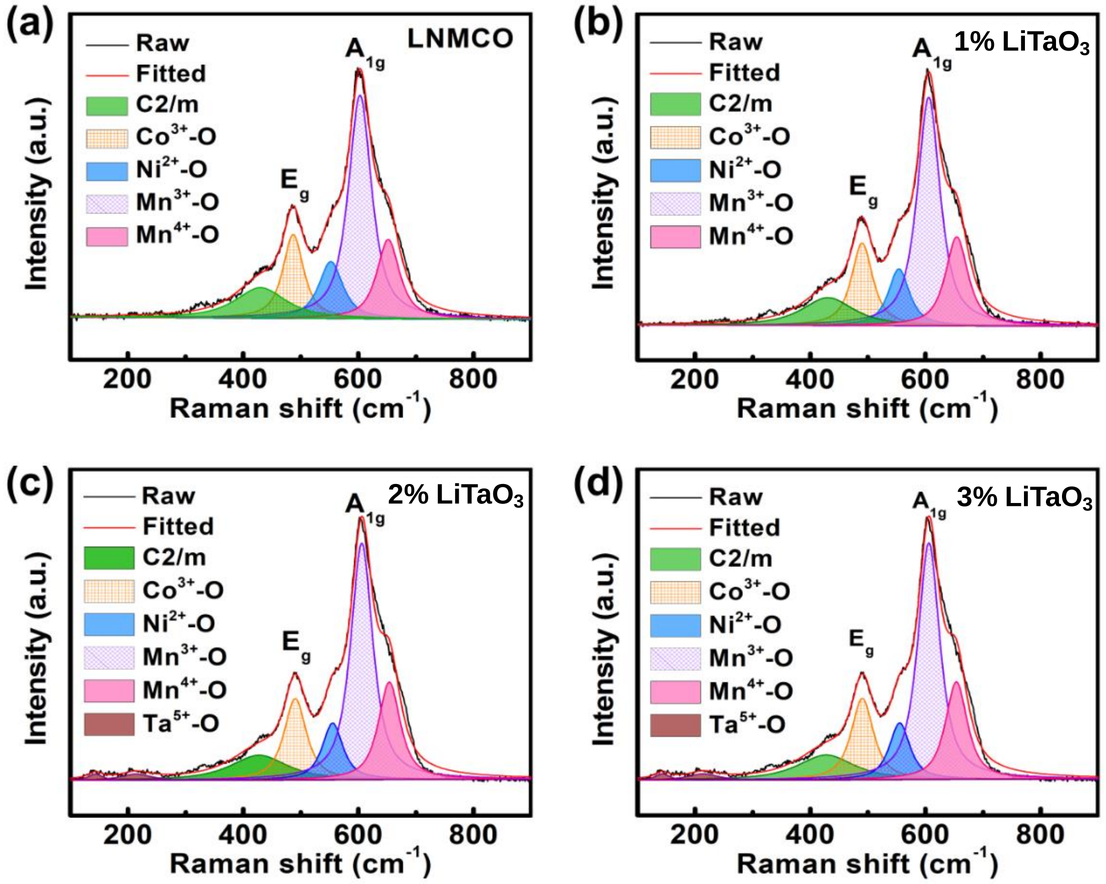

Figure S7 Raman fitting results of **(a)** LNMCO, **(b)**1%, **(c)** 2% and **(d)** 3% $LiTaO_3$ samples.

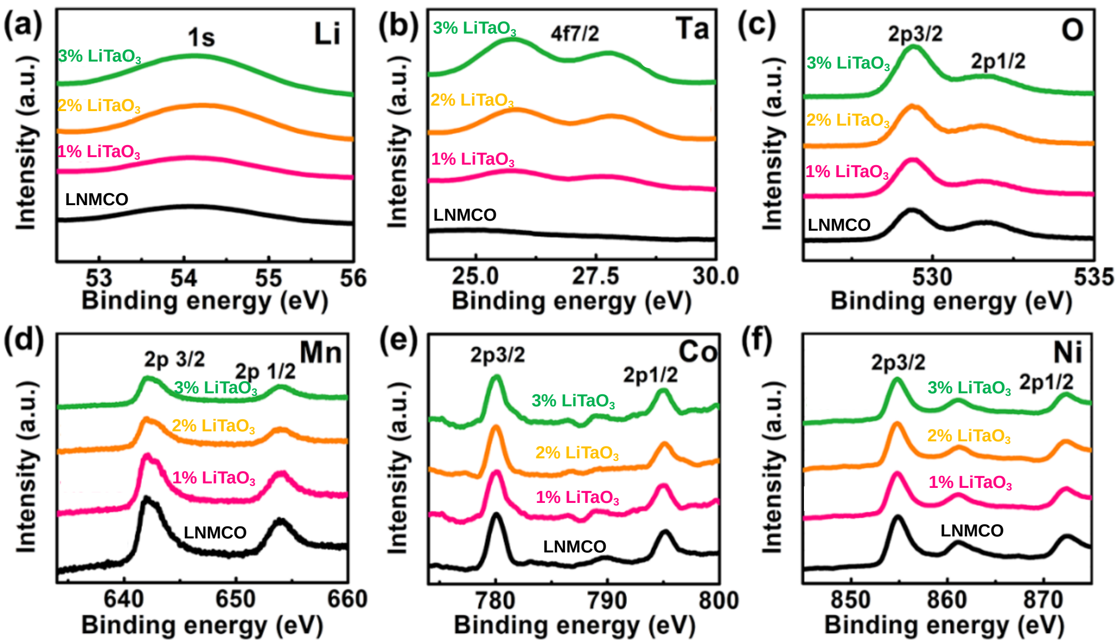

Figure S8 XPS spectra for **(a)** Li, **(b)** Ta, **(c)** O, **(d)** Mn, **(e)** Co and **(f)** Ni in the pristine LNMCO, 1 %, 2% and 3% $LiTaO_3$ samples.

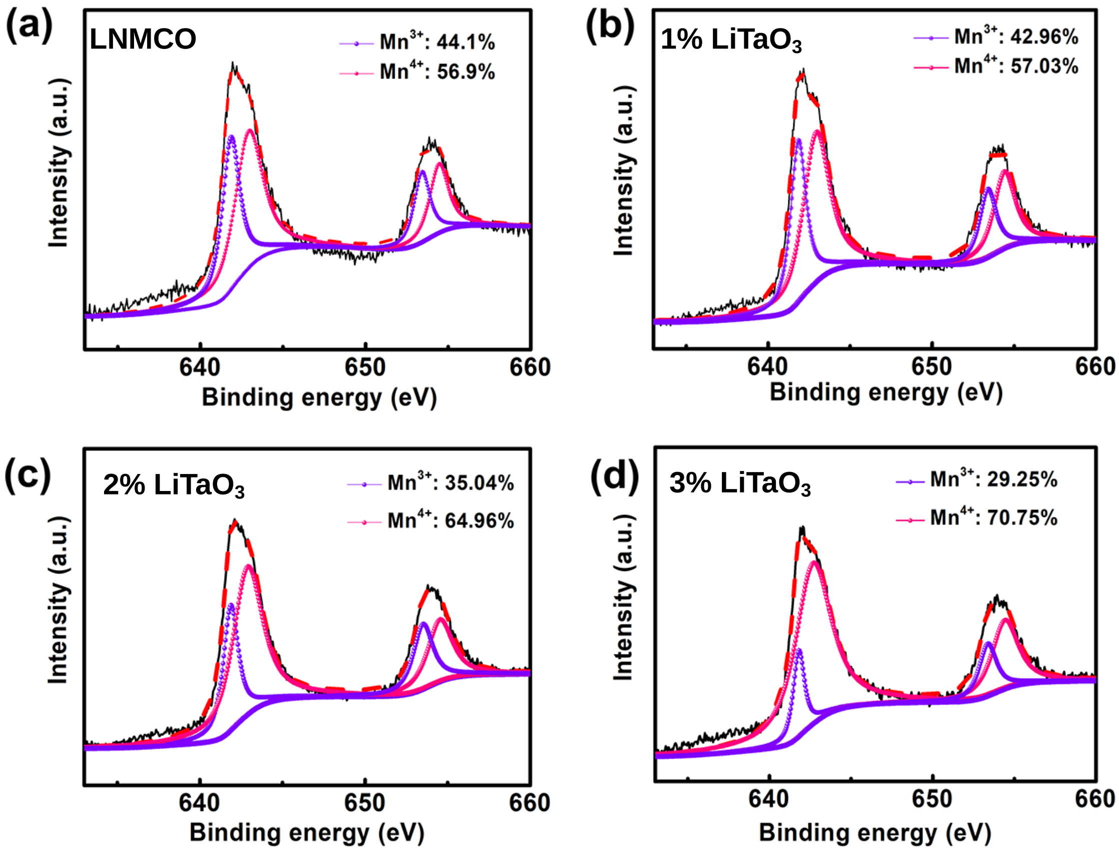

Figure S9 Fitting XPS spectra of Mn element in **(a)** LNMCO, **(b)** 1%, **(c)** 2 % and **(d)** 3 % $LiTaO_3$ samples.

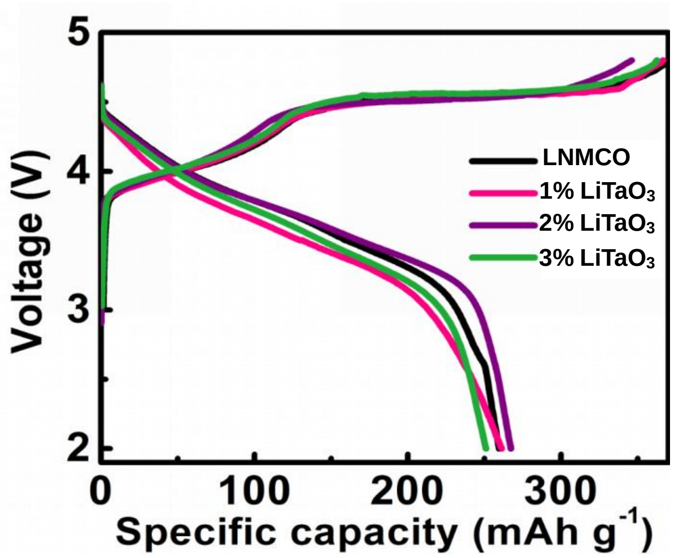

Figure S10 Charge and discharge profiles of LNMCO before and after $LiTaO_3$ modification.

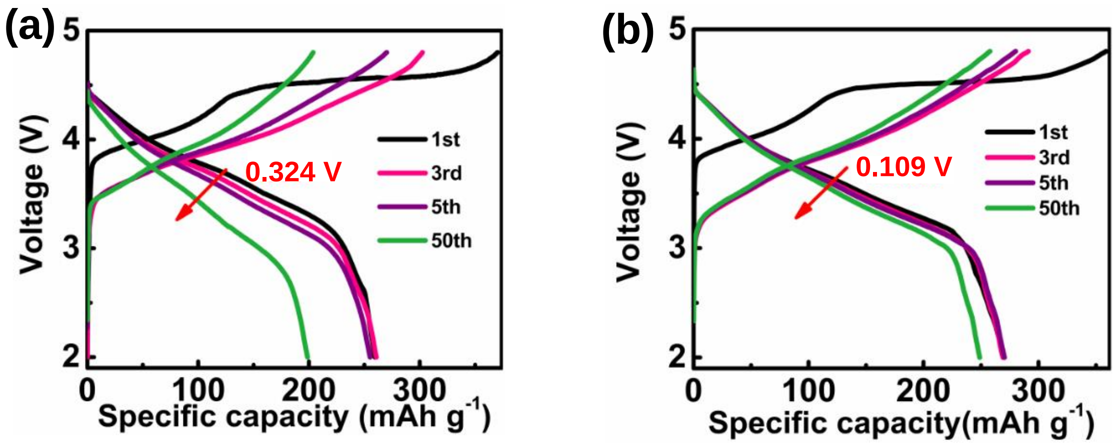

Figure S11 Voltage declining curves of **(a)** LNMCO and **(b)** 2% $LiTaO_3$ samples at the different cycles.

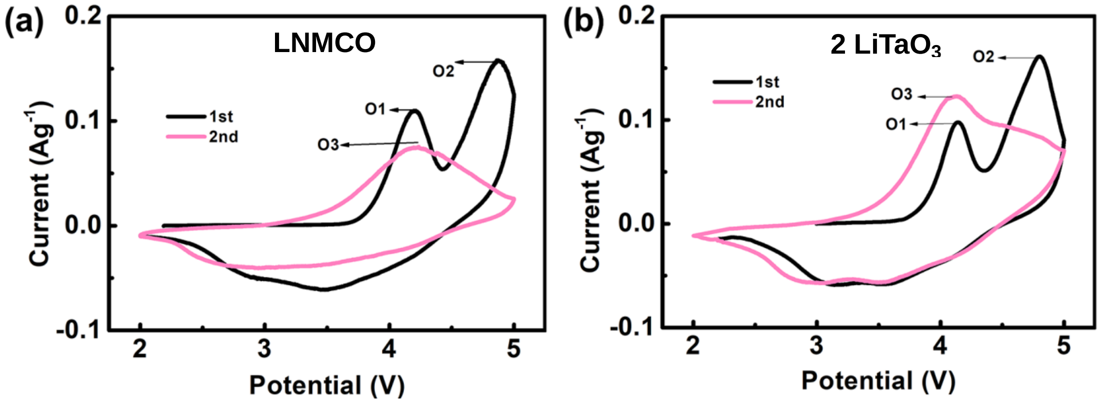

Figure S12 Cycle voltammetry curves (CV) of the **(a)** pristine LNMCO and **(b)** the 2 % $LiTaO_3$ samples under the 1st and 2nd cycle.

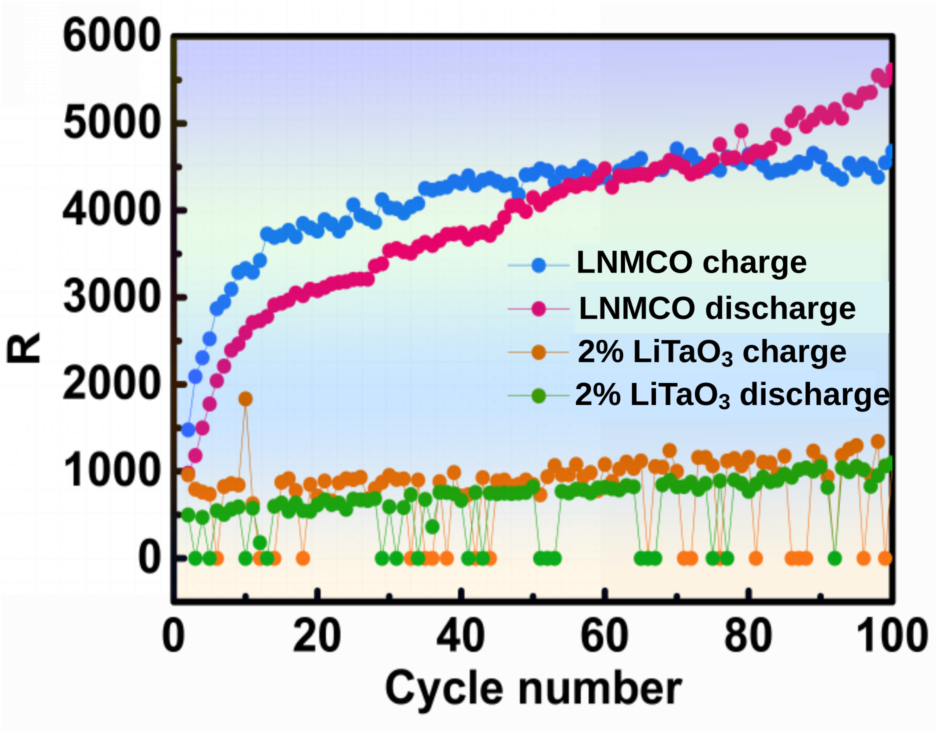

Figure S13 DC internal resistance of charging and discharging of pristine LNMCO and 2 % $LiTaO_3$.

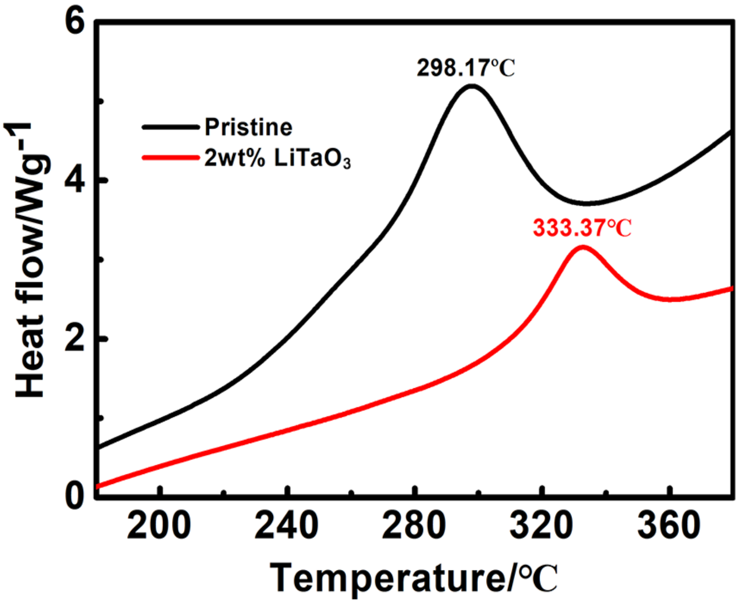

Figure S14 DSC curves of the pristine LNMCO and the 2 % $LiTaO_3$ samples.

For the practical application of LIBs, safety is a critical concern, especially for high energy density devices. The thermal stabilities of pristine LNMCO and 2 % $LiTaO_3$ electrodes were investigated by DSC measurement, as shown in **Fig. S12**, in which the exothermic peak of 2 % $LiTaO_3$ material shifts to a higher temperature (333 ^{o}C) than that of the pristine LNMCO (298 ^{o}C). Meanwhile, the peak area of the 2 % $LiTaO_3$, on behalf of heat release, distinctly reduces compared with that of the pristine LNMCO. Therefore, it could be concluded that the thermal stability of 2 % $LiTaO_3$ is significantly enhanced with $LiTaO_3$-coating layer.

Table S1 Lattice parameters and d-spacing values of the main diffraction peaks for $LiTaO_3$ and LNMCO.

	**a (Å)**	**c (Å)**	**d (nm)**
**LNMCO**	2.852(2)	14.226(2)	0.47
**$LiTaO_3$**	5.154(3)	13.783(5)	0.37

The lattice mismatch is calculated according to the formula as following:

$$\delta = \frac{a_{LNMCO} - a_{LTO}}{a_{LNMCO}}$$

here a_{LNMCO} represents the lattice parameter of the pristine LNMCO material, and a_{LTO} represents the lattice parameter of the $LiTaO_3$ coating material.

According to the structure diagrams of LNMCO and $LiTaO_3$ as shown in **Fig. S3**, the most preferred orientation is paralleled with //aob. In addition, *a* in the above formula refers to cell parameter in the crystal unit. According to the collected XRD profiles and thus calculated lattice constants (shown in Table S1), the epitaxial growth of $LiTaO_3$ has a great chance to be stacked along *c* orientation due to the low lattice mismatch.

Table S2 Calculated lattice parameters from refinement results of pristine and $LiTaO_3$-LNMCO samples.

Sample	a(Å)	c(Å)	$I_{(003)}/I_{(104)}$	c/a
LNMCO	2.852(2)	14.226(2)	1.424	4.988
1 % $LiTaO_3$	2.852(3)	14.227(4)	1.429	4.988
2 % $LiTaO_3$	2.852(5)	14.228(2)	1.439	4.989
3 % $LiTaO_3$	2.852(6)	14.229(6)	1.423	4.989

Table S3 The calculated values of FWHMs and polarization degree from CV spectra of the pristine LNMCO and 2% $LiTaO_3$ electrodes.

Sample	FWHM			Polarization		
	O1	O2	O3	O1	O2	O3
LNMCO	0.411	0.378	0.657	1.341	1.386	1.599
2% $LiTaO_3$	0.369	0.365	0.415	0.992	1.197	1.155

Table S4 R_s, R_{sf}, R_{ct} of pristine LNMCO and 2 % $LiTaO_3$ at different cycles.

		1st	10th	20th	30th	40th
LNMCO	R_s	2.0	3.4	4.7	6.9	10.4
	R_{sf}	25.0	26.1	35.1	55.8	131.2
	R_{ct}	467.0	216.8	447.7	608.6	825.0
2 % $LiTaO_3$	R_s	1.3	2.9	2.5	3.0	3.2
	R_{sf}	15.2	16.9	17.4	18.9	28.9
	R_{ct}	127.2	195.6	193.2	209.0	261.4

Table S5 The elastic coefficients of the pristine LNMCO sample.

	**Elastic coefficient (MPa)**
	75596
	75272
	77576
	77351
**LNMCO**	75189
	74087
	76784
	77125
	77825
**Mean**	76312
